# Assessment of the risk posed to Singapore by the emergence of artemisinin-resistant malaria in the Greater Mekong Subregion

**DOI:** 10.5365/wpsar.2018.9.2.011

**Published:** 2019-05-16

**Authors:** Emma Xuxiao Zhang, Jean-Marc Chavatte, Cherie See Xin Yi, Charlene Tow, Wong Jia Ying, Kamran Khan, Olivia Seen Huey Oh, Sarah Ngeet Mei Chin, Khong Wei Xin, Zubaidah Said, Lyn James, Jeffery Cutter, Marc Ho, Jeannie Su Hui Tey

**Affiliations:** a Ministry of Health, Singapore.; b National Public Health Laboratory, National Centre for Infectious Diseases, Singapore.; c Li Ka Shing Knowledge Institute, St. Michael’s Hospital, Toronto, Canada.; d Department of Medicine, Division of Infectious Diseases, University of Toronto, Toronto, Canada.

## Abstract

**Objective:**

To assess the public health risk to Singapore posed by the emergence of artemisinin-resistant (ART-R) malaria in the Greater Mekong Subregion (GMS).

**Methods:**

We assessed the likelihood of importation of drug-resistant malaria into Singapore and the impact on public health of its subsequent secondary spread in Singapore. Literature on the epidemiology and contextual factors associated with ART-R malaria was reviewed. The epidemiology of malaria cases in Singapore was analysed. The vulnerability and receptivity of Singapore were examined, including the connectivity with countries reporting ART-R malaria, as well as the preparedness of Singaporean health authorities. Sources of information include international journals, World Health Organization guidelines, data from the Singapore Ministry of Health and National Public Health Laboratory of the National Centre for Infectious Diseases, and the International Air Transport Association.

**Results:**

The importation of ART-R malaria into Singapore is possible given the close proximity and significant travel volume between Singapore and the GMS countries reporting artemisinin resistance. Singapore’s vulnerability is further enhanced by the presence of foreign workers from neighbouring endemic countries. Nonetheless, the overall likelihood of such an event is low based on the rarity and decreasing trend of imported malaria incidence.

**​**

With the presence of *Anopheles* vectors in Singapore, imported cases of drug-resistant malaria could cause secondary transmission. Nevertheless, the risk of sustained spread is likely to be mitigated by the comprehensive surveillance and control system in place for both infected vectors and human cases.

**Discussion:**

This risk assessment highlights the need for a continued high degree of vigilance of ART-R malaria locally and globally to minimize the risk and public health impact of drug-resistant malaria in Singapore.

## Objective

Despite remarkable global progress in the fight against malaria since 2010, growing resistance to antimalarial drugs remains the biggest challenge on the path towards malaria elimination. (*1*) To date, drug resistance has been recorded in *Plasmodium falciparum, P. vivax* and *P. malariae.* (*2*, *3*) The development of resistance by *P. falciparum* to nearly all antimalarial drugs, including current first-line treatments artemisinin and its derivatives, has become an issue of utmost concern. The Greater Mekong Subregion (GMS), which comprises Cambodia, the Lao People's Democratic Republic, Myanmar, Thailand, Viet Nam and Yunnan Province of China, has long been the epicentre of antimalarial drug resistance. (*4*) The first cases of artemisinin resistance were reported in Cambodia in 2008. (*5*) Since then, artemisinin resistance has been observed in other countries in the GMS and in neighbouring India. (*5*, *6*) Concomitantly, variable levels of resistance to the partner drugs used in artemisinin-based combination therapies (ACTs) have been reported. (*4*)

Singapore is a globally connected city-state in South-East Asia with high travel connectivity with many countries in the world, including those in the GMS, due to its position as a travel and trade hub. Although Singapore has been declared malaria-free by the World Health Organization (WHO) since 1982, Singapore is at risk of importation of emerging diseases including artemisinin-resistant (ART-R) malaria. In view of the emergence of resistance across the GMS and the spread beyond its borders, we carried out an analysis to assess the risk of importation and secondary spread of ART-R malaria infection in Singapore.

## Methods

The risk of importation of drug-resistant malaria into Singapore and the public health impact of its subsequent secondary spread in Singapore were assessed following WHO guidance on the risk assessment of acute public health events. (*7*) The process of risk assessment included relevant literature review, epidemiological analysis of malaria cases in Singapore, analysis of the air travel volume between Singapore and countries reporting artemisinin resistance, an assessment of the vulnerability and receptivity of Singapore, and the preparedness of Singaporean health authorities to a potential (case/introduction/outbreak) of ART-R malaria. The risk assessment was conducted by four public health officers specializing in public health surveillance, epidemiology and risk analysis of infectious diseases. Their findings were reviewed by a broader group of experts from the Singapore Ministry of Health (health ministry) in the areas of public health, laboratory medicine, epidemiology, infectious diseases, risk communication and emergency preparedness and response.

The epidemiology of malaria cases in Singapore was analysed based on information released by the health ministry. (*8*) A qualitative review of the public health measures taken by Singapore in response to the emerging threat was conducted based on information released by the health ministry and the Singapore National Environment Agency (NEA). The volume of travellers on commercial flights originating from countries with ART-R malaria and with final destinations in Singapore in 2017 was analysed using data from the International Air Transport Association (IATA) in Singapore. The IATA data are reported monthly and contain anonymized, itinerary-level passenger volumes. The data capture an estimated 90% of the world’s air traffic, with the remainder being imputed using market intelligence. Full itineraries of the travellers have been used, including the initial airport of embarkation, final destination and any connecting flights. IATA data have been used previously to inform risk assessments of the spread of pathogens of epidemic potential. (*9*) Among the imported *P. falciparum* cases, the monitoring of artemisinin resistance based on the Kelch 13 (K13) gene was performed by the Malaria Reference Centre from the National Public Health Laboratory (MRC-NPHL) by polymerase chain reaction (PCR) amplification and sequencing according to the protocol of Ariey et al. (*10*)

## Risk assessment

### Hazard assessment

Malaria is caused by *Plasmodium* parasites that are transmitted to people through the bites of female *Anopheles* mosquitoes with infectious sporozoites of malaria parasites. (*11*) Five *Plasmodium* species cause malaria in humans: *P. falciparum*, *P. vivax*, *P. malariae*, *P. ovale* and *P. knowlesi*. *P. falciparum* is responsible for most malaria-related deaths globally and is the most prevalent malaria parasite in Africa, while *P. vivax* is the dominant malaria parasite in most countries outside of subSaharan Africa including those in South-East Asia. (*1*) The first symptoms of malaria—fever, headache, chills and vomiting—usually appear between 10 to 15 days after a bite from a vector mosquito with infectious sporozoites of malaria parasites. Without prompt diagnosis and treatment, *P. falciparum* malaria can rapidly progress to severe illness and death. (*11*) Currently, the most effective treatments are ACTs. (*12*, *13*)

Drug resistance has been one of the greatest challenges in fighting malaria. Resistance usually develops progressively, from the initial delay of parasite clearance in a few locations to the gradual expansion of geographic range and increase in prevalence, eventually leading to treatment failure. Of the various antimalarial drugs available, chloroquine was the agent of choice for many years because of its safety, efficacy and affordability. However, since its first detection along the border of Cambodia and Thailand in 1957, resistance of *P. falciparum* to chloroquine has spread to almost everywhere that *P. falciparum* exists. (*14*) *P. falciparum* has also developed resistance to nearly all other available antimalarial drugs, including sulfadoxine, pyrimethamine, amodiaquine, mefloquine, piperaquine, atovaquone and an increasing frequency of reported quinine resistance in several regions. (*15*)

The first clinical evidence of artemisinin resistance originated in western Cambodia in 2008; however, further retrospective studies of molecular markers have indicated that artemisinin resistance likely emerged before 2001 and the widespread usage of ACTs. (*5*, *16*, *17*) The extensive and often suboptimal usage of monotherapies as well as the genetic background of parasites in the GMS were thought to have contributed to the development of resistance. (*18*) Since then, emergence of resistance to artemisinin and ACT partner drugs such as piperaquine have been reported in other areas in the GMS. (*19*, *20*) Nevertheless, most patients with delayed parasite clearance can still be cured using ACTs as long as the partner drug remains effective; there is no evidence that higher levels of artemisinin resistance (full resistance) have emerged. (*4*)

### Exposure assessment

#### Reports of imported malaria infections in Singapore

In Singapore, malaria was the most common vector-borne disease in the early 20th century, resulting in substantial morbidity and mortality. (*21*) With strengthened epidemiological and vector control measures, Singapore was certified malaria-free by WHO in November 1982. Since 2010, the annual incidence of malaria has continued to decrease from 3.7 cases per 100 000 population in 2010 to 0.7 cases per 100 000 population in 2017. Incidence has been maintained below 1 per 100 000 population in recent years. The number of imported malaria cases steadily decreased from 187 cases in 2010 to 39 cases in 2017.

Among the 290 cases of malaria reported in Singapore between 2013 and 2017, 289 (99.7%) were imported cases, of whom 141 (49%) were work-permit or employment pass holders. The majority of imported cases were from South-East Asia (16%), Africa (16%) and India (62%). Among the cases imported from South-East Asia, most were from Indonesia and Malaysia, which accounted for 8% and 4% of cases, respectively. Most of the cases of malaria reported in Singapore between 2013 and 2017 were caused by *P. vivax* (70%) followed by *P. falciparum* (22%).

In a retrospective survey from 2008 to 2017, 12 out of 209 (5.7%) *P. falciparum* cases tested had mutations possibly associated with artemisinin resistance (**Table 1**). Of the 12 cases, three cases had validated K13 resistance mutations while two cases had candidate K13 resistance mutations classified by WHO. (*4*) However, none experienced treatment failure and all recovered without complications.

**Table 1:** Number of *P. falciparum* malaria cases in Singapore with mutations possibly associated with artemisinin resistance detected by K13 molecular marker analysis

Years	No. of notified cases	No. of tested cases*	Wild types^‡^	Mutants			
				SNPs^^^	Numbers	Origins	GenBank accession numbers
2008	37	20	18	F446I	1	Myanmar	MH341694
				N412T, D452E, V454A, K503K^#^	1	India	MH341695 MH341696^#^
2009	33	24	22	C580Y Y630Y	1 1	Myanmar Indonesia	MH341697 MH341698
2010	55	52	46	F446I G453C N458D R575K	1 2 1 2	Myanmar Myanmar Ghana Myanmar	MH341703 MH341701/MH341704 MH341700 MH341699/MH341702
2011	25	25	22	G453C S623N	2 1	Myanmar Malaysia	MH341705/MH341706 MH341707
2012	28	28	28	-			
2013	21	21	21	-			
2014	9	8	8	-			
2015	13	12	12	-			
2016	3	2	2	-			
2017	18	17	15	C469C	2	Ghana United Republic of Tanzania	MH341708 MH341709
**Total**	**242 (100%)**	**209** **(86.4%)**	**194 (92.8%)^‡^**	**15 mutants (7.2%)** **3 synonymous (1.4%)** **12 non-synonymous (5.7%)**			

SNP: single nucleotide polymorphism.

* Not all notified cases were tested due to unavailability of samples.

‡ All wild-type sequences were identical to ***P. falciparum*** 3D7 control strain sequence: MH341710.

# Case with a mixed ***P. falciparum*** strain infection harbouring an alternative synonymous mutation: V454V.

^ Non-synonymous mutations are bolded. Double and single underlined variants represent validated and associated (without statistical significance) K13 resistance mutations, respectively, according to WHO. (*4*) Non-underlined variants represent other mutations detected but non-evaluated by WHO. (*4*)

#### Travel volume from the GMS to Singapore

Among cities outside the GMS, Singapore receives the highest number of travellers from the GMS (**Table 2A**). From 2013 to 2017, there was an average of more than 303 000 travellers each month from the GMS to Singapore (**Table 2B**). Thailand accounted for the highest number of travellers followed by Viet Nam and Myanmar. More than 15 000 monthly travellers to Singapore came from Cambodia where artemisinin resistance was first detected. Despite being a major international transportation hub with high connectivity to the GMS, importation of malaria into Singapore appears to be rare (**Table 2B**).

**Table 2:** Connectivity between Singapore and the GMS countries

A. Top 10 final destination cities outside the GMS for travellers originating from countries/areas with artemisinin-resistant malaria in the GMS, 2017				
Rank	Final destination city	Final destination country	Total travel volume received	% of travellers
1	Singapore	Singapore	4 012 918	6.2
2	Seoul	Republic of Korea	3 652 737	5.6
3	Shanghai	China	3 006 325	4.6
4	Hong Kong Special Administrative Region	China	2 865 969	4.4
5	Guangzhou	China	2 608 468	4.0
6	Beijing	China	2 467 999	3.8
7	Kuala Lumpur	Malaysia	2 413 439	3.7
8	Chengdu	China	2 144 415	3.3
9	Tokyo	Japan	1 842 503	2.8
10	Chongqing	China	1 667 531	2.6

***Source:*** data were obtained from the International Air Transport Association.

**Table:** 

B. Number of malaria cases imported into Singapore from 2013 to 2017 from GMS countries/areas in relation to travel volume					
Rank	Origin country/area	No. of imported cases from 2013 to 2017	Total travel volume received from 2013 to 2017	Average monthly travel volume received from 2013 to 2017	% of total traveller volume
1	Thailand	2	11 261 683	187 695	61.9%
2	Viet Nam	0	4 198 163	69 969	23.1%
3	Myanmar	8	1 484 219	24 737	8.1%
4	Cambodia	0	905 564	15 093	5.0%
5	Yunnan (China)	2*	227 237	3787	1.2%
6	Lao People's Democratic Republic	0	120 626	2010	0.7%
**Total**		**12**	**18 197 492**	**303 292**	**100%**

* The two cases were imported from China, but information on the provinces where the cases were from was not available.

### Context assessment

#### Vector distribution

Singapore remains vulnerable and receptive to the reintroduction of malaria and the introduction of drug-resistant malaria due to the presence of *Anopheles* vectors. Among the over 400 species of *Anopheles* mosquitoes discovered globally to date, about 70 species are potential vectors of malaria. (*22*) In Singapore, the two most common *Anopheles* species are *Anopheles epiroticus* and *Anopheles sinensis*. (*23*) *Anopheles epiroticus*, *Anopheles maculatus* and *Anopheles letifer*, were reported to be involved in malaria outbreaks in Singapore in the 1960s and 1970s, while *Anopheles sinensis* was implicated in the 2009 outbreak. (*24*, *25*)

#### Preparedness and response in Singapore

Malaria surveillance and control in Singapore is under the purview of two public health agencies: the NEA, which undertakes the surveillance and control of *Anopheles* mosquitoes, and the Singapore health ministry, which is responsible for case surveillance and epidemiological investigation. Vector control remains the cornerstone for controlling mosquito-borne diseases, including malaria. NEA has put in place an integrated vector surveillance and control programme comprising environmental management and source reduction. (*26*) NEA has also identified specific malaria receptive areas for regular *Anopheles* surveillance and control. (*27*) Malaria is a legally notifiable disease under the Infectious Disease Act and all medical practitioners and laboratories are required to notify the health ministry within 24 hours of diagnosis. (*28*) The health ministry then investigates all cases of malaria to determine if transmission is likely to be locally acquired or imported and assess whether any clusters are present. The MRC-NPHL has the capability to detect a large panel of molecular markers associated with antimalarial drug resistance, including the WHO-recommended K13 gene (as a marker of artemisinin resistance) and several genes associated with partner drug resistance.

To address the issue of imported malaria cases among foreign workers, Singapore implemented compulsory screening for malaria for foreign workers in 1997 as part of the pre-employment medical examinations. Among Singapore residents diagnosed with imported malaria infections from 2012 to 2016, more than 90% did not observe adequate preventive measures such as taking chemoprophylaxis before overseas travel. (*29*) Such behaviours could be due to the lack of risk perception associated with travel, especially within Asia, and the lack of awareness of travel medicine among travellers. (*29*)

#### Past outbreaks of malaria in the event of an imported case

Although imported cases continue to pose challenges for malaria control, the chances of resumption of endemic transmission are small as elimination tends to be a stable state. (*30*) Singapore has maintained its malaria-free status (*31*) since 1982. Between 1983 and 2009, 32 outbreaks involving 225 cases were reported, and the majority of the cases were imported through foreign workers with relapsing *P. vivax* malaria. (*26*) Further transmissions from these occasional outbreaks were promptly curbed by aggressive preventive and remedial actions, including extensive vector surveillance and control measures; early case detection through blood and fever surveys in malaria receptive areas; and risk communication to medical practitioners as well as health education for the public. (*26*) No outbreaks have been reported since 2010.

#### Measures taken by GMS countries

To prevent global spread of artemisinin resistance, containment efforts have been initiated in the GMS. In 2015, WHO launched the *Strategy for malaria elimination in the Greater Mekong Subregion (2015–2030)*, which was endorsed by all GMS countries. (*31*) All GMS countries have begun to implement national malaria elimination strategies that have resulted in a significant reduction in malaria cases and death; the surveillance of the efficacy of antimalarial drugs has led to prompt updating of malaria treatment policies in most GMS countries. (*1*)

### Risk characterization

The risk imposed to Singapore by the emergence of ART-R malaria was characterized using the information collected; key factors were considered to assess the likelihood of importation of cases into Singapore and the impact on public health (**Table 3**).

**Table 3:** Risk characterization matrix for the public health risk posed to Singapore

	Hazard	Exposure	Context
Potential for importation of ART-R malaria into Singapore	Artemisinin resistance is defined as delayed parasite clearance following treatment with an artesunate monotherapy or ACT. This represents partial resistance. Since its first detection in Cambodia in 2008, artemisinin resistance in *P. falciparum* has been found in the GMS and has spread to neighbouring India.	A long incubation period of malaria ranging from 7 to 30 days means that international travel of infected persons in incubation period is possible. Cases of recrudescence can also be imported. Imported cases were 99.7% of the malaria cases reported in Singapore between 2013 and 2017. Close connectivity between GMS countries and Singapore; an average of 303 292 travellers, come from the GMS every month. From 2008 to 2017, 12 out of 209 (5.7%) *P. falciparum* malaria cases imported to Singapore had mutations possibly associated with artemisinin resistance; none experienced treatment failure and all recovered without complications.	Singapore has foreign workers from neighbouring endemic countries who contribute to imported malaria cases in Singapore. All GMS countries have begun to implement national malaria elimination strategies that have resulted in significant reduction of malaria cases and deaths. The effectiveness of the strategies in stopping the spread of artemisinin resistance need to be closely monitored.
Potential public health impact to Singapore	Infection results in acute febrile illness. The first symptoms may be mild and difficult to recognize. Without treatment within 24 hours, *P. falciparum* malaria can progress to severe illness, often leading to death. To date, artemisinin resistance has not been associated with increased morbidity or mortality.	The majority of the population in Singapore are susceptible to malaria. Some population groups are at higher risk of infection and developing severe diseases, including infants, children under 5 years of age, pregnant women, patients with HIV/AIDS, and non-immune migrants, mobile populations and travellers.	The vector for malaria, *Anopheles* mosquitoes, is one of the most common mosquito genera present in Singapore. Comprehensive surveillance and control systems for both vectors and human cases are in place in Singapore. Policies are in place to prevent the importation of malaria by foreign workers; increased awareness for personal protective measures is needed among local residents travelling to malaria-endemic regions.

ACT: artemisinin-based combination therapy; ART-R: artemisinin-resistant; GMS: Greater Mekong Subregion.

The risk characterization of likely based on the likelihood of importation and the according minimal consequence suggest that the overall risk of ART-R malaria to Singapore is low. The importation of a case of ART-R malaria into Singapore is possible given the close proximity and significant travel volume between Singapore and the GMS countries reporting artemisinin resistance. Singapore’s vulnerability is further enhanced by the presence of foreign workers from neighbouring endemic countries. Nonetheless, the overall likelihood of importation is considered low based on the rarity and the decreasing trend of imported malaria incidence over the past few years.

With the presence of *Anopheles* vectors in Singapore, imported cases of ART-R malaria can cause secondary transmission. The risk of sustained spread is likely to be mitigated by the comprehensive surveillance and control system in place for both infected vectors and human cases as observed in the past local outbreaks of malaria initiated by imported cases.

## Discussion

Singapore is the top destination for travellers from the GMS. Among the imported *P. falciparum* cases in Singapore, 5.8% had genetic mutations that may confer resistance to artemisinin. The presence of competent local vectors, the high volume of travel from regions with ART-R malaria, and the presence of foreign workers from neighbouring endemic countries make it possible that drug-resistant malaria could be imported and introduced to Singapore. To reduce the risk of Singapore residents acquiring malaria infections overseas, pre-travel health education, particularly by travel agents, the media and health-care providers, can increase awareness of the risk of contracting malaria overseas so that personal preventive measures can be taken.

Secondary spread following an imported case is also possible. However, any spread is not likely to be sustained. Malaria has not re-established itself as an endemic disease in Singapore despite local outbreaks since it was declared malaria-free in 1982. The Singapore health ministry and NEA have implemented comprehensive malaria surveillance and control programmes to detect cases and curb the transmission of local outbreaks.

The risk assessment has some limitations. The assessment is based on limited data as the number of imported cases of malaria in Singapore is small. As the risk characterization was defined by the epidemiological and contextual knowledge available currently, conclusions could change as new information emerges. Ongoing studies on genetic mutations, particularly their underlying molecular and cellular mechanisms and their phenotypic manifestations in resistance, could provide a better understanding of an epidemic and facilitate the design of surveillance and control measures. Identification of new molecular markers and improvements in laboratory capability continues to impact disease surveillance as illustrated by the significant progress in global surveillance of artemisinin resistance expedited by the discovery of the molecular marker K13. (*12*) Risk assessment will also change as new treatment options become available. Even though an assessment of the risks posed by resistance to partner drugs in the ACTs is out of scope for this paper, we recognize the risk of such resistance and the importance of monitoring all molecular markers of antimalarial-drug resistance. Analysis is currently under way to test molecular markers for partner drugs and assess potential variations. In addition, artemisinin resistance has also been observed in non-GMS countries, including countries in Africa, although the occurrence is very rare. (*4*) The risk of importation of ART-R malaria from these non-GMS countries is not discussed in this paper because of low connectivity of Singapore with these countries. Nevertheless, it warrants our close monitoring of the development of global situations.

In conclusion, in view of the emergence of ART-R malaria in the GMS and its geographical expansion, this risk assessment highlights the need for a high degree of vigilance over the local and global situation to be maintained to minimize the risk and severity of the public health threat of ART-R malaria to Singapore.
